# Raising the Guanosine-Based Molecules as Regulators of Excitable Tissues by the Exosomal-Vehiculated Signaling

**DOI:** 10.3389/fphar.2021.658370

**Published:** 2021-07-30

**Authors:** Tiziana Pietrangelo

**Affiliations:** ^1^ Department of Neuroscience, Imaging and Clinical Sciences, University “G. D’Annunzio” of Chieti-Pescara, Chieti, Italy; ^2^ Interuniversity Institute of Myology, Chieti, Italy

**Keywords:** guanosine, neopterin, skeletal muscle, satellite cells, exosomes

## Introduction

Human satellite cells are able to secrete exosomes containing guanosine-based molecules, mainly guanosine (Guo) (Pietrangelo et al., 2018). This evidence suggests that Guo-based exosomal cargo could have an important role in the secretome’s fascinating world. It may be that exosomes stuffed with Guo released by human skeletal muscle stem cells communicate with surrounding fibers (paracrine action) and with stem cells (autocrine action) for muscle regeneration as for the endocrine function. Till now, a large number of studies in the last decade have shown that striated muscle contraction indeed has an endocrine function. This documented endocrine activity is based on the release of proteins and peptides called “myokines”; however, the finding of Guo-stuffed exosomes and GTP in the extracellular milieu envisages a new scenario in the skeletal muscle–dependent communication.

Interestingly, the Guo-exosomal cargo released by muscle compartment could be delivered for the central nervous system, in which the Guo-based molecules have been demonstrated to act in different physiological and pathological conditions (Bettio et al., 2016). Our unpublished data reveal that Guo-stuffed exosomes are present in human blood and have also been found in urine. So, despite the general idea that Guo-stuffed exosomes are released for the endocrine purpose has to be confirmed, it seems fairly possible. Recently, several studies have reported the involvement of Guo-mediated signaling in behavioral outcomes, such as neuroprotection, and anxiolytic-like and analgesic effects (Almeida et al., 2017; Frinchi et al., 2020).

Yet, Guo-based molecules must surely have an important role in signal transduction if excitable cells preserve plasma membrane–specific receptor binding sites, as demonstrated for the extracellular guanosine triphosphate (eGTP) both in murine myoblasts and rat neuron-like cells (Mancinelli et al., 2012; Mancinelli et al., 2020). In fact, eGTP stimulation has a role in myogenesis, *via* activation on myoblast plasma membranes, opening the intermediate calcium-activated potassium channels on the plasma membrane. The resulting transient cellular hyperpolarization acts as a first “electric shock” for myoblasts that soon would accelerate proliferation and become committed, partly to rescue quiescence, partly to fusion and differentiation process (Pietrangelo et al., 2002; Pietrangelo et al., 2006). Moreover, GTP binding sites are present also in PC12 cells, differentiated as sympathetic like cells (Gysbers et al., 2000), as well as in SH-SY5Y (Guarnieri et al., 2009) and for Guo in rat brain membranes (Traversa et al., 2002; Traversa et al., 2003; Zuccarini et al., 2018).

However, the source of eGTP has to be deeply investigated. Apart from the Guo exosomal cargo, the release of GTP from synaptic vesicles and astrocytes in central nervous system (Tasca et al., 2018) has also been demonstrated. On this trail, it is not a pilgrim to think that motoneurons and/or Schwann cells also could release GTP in skeletal muscle following the firing of action potentials. Moreover, considering that mouse myoblasts showed intracellular calcium increase also in response to extracellular Guo, it could also be argued that eGTP is hydrolyzed by ectonucleotidases to extracellular guanosine responsible for activating the extracellular binding sites. Despite many doubts about the source of GTP and Guo in the extracellular milieu of skeletal fibers, most probably Guo-based molecules derive from fiber damage.

Briefly, although Guo-based purines have been largely demonstrated as extracellular signaling molecules with important functions in the central nervous system, the idea that skeletal muscle cells sustain this signaling remained subdued.

My opinion is that Guo-based purines are regulators, produced by skeletal muscle contraction, released as exosomal cargo, and delivered to the nervous system compartment in order to regulate and positively influence it.

**GRAPHICAL ABSTRACT:**
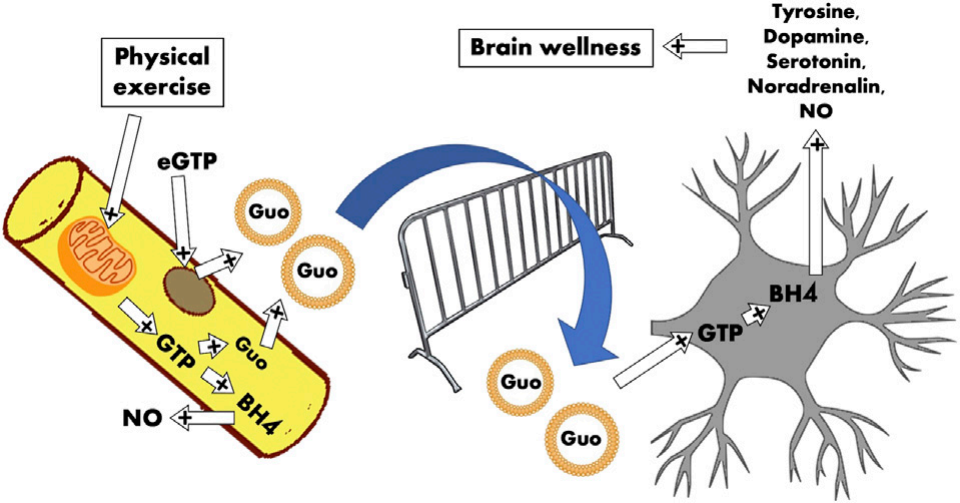
The cartoon represents the skeletal muscle compartment that releases guanosine exosomal‐vehiculated regulators for the neuronal compartment. The idea is that the satellite cells (brown circle) and skeletal fibers (yellow) are the continuous source of Guo‐based molecules able to exert positive regulation on the central nervous system. The contraction, responsible for mitochondrial GTP production through GDP phosphorylation by means of succinyl‐CoA synthetase, sustains GTP and in turn Guo disponibility and BH4 synthesis. Guo‐stuffed exosomes are delivered to neurons that receive adequate amount of Guo‐based molecules to transform into BH4 and in turn to several neurotransmitters (dopamine, serotonin, and noradrenaline).

### The mitochondrial activity is a conspicuous contributor for iGTP level that, in turn, could promote the Guo-exosomal cargo

In this scenario, the GTP and Guo-exosomal cargo release by skeletal cells need to be switched on by some signal specifically attributable to skeletal muscle. Maybe it is linked in some way to contraction? If contraction continuously supplies GTP, maybe the cells sense enough amount of Guo-based molecules and decide to secrete it, mainly as Guo stuffed into exosomes and/or as free GTP.

It is worth reminding that there is a conspicuous GTP production selectively linked to mitochondrial metabolism *via* the GDP phosphorylation by means of succinyl-CoA synthetase on mitochondrial succinyl-CoA transformation in succinate.

Briefly, one acetyl-CoA (final malate) produces three NADH, one FADH, one GTP, and two CO_2_. Noteworthy, the malate concentration in the mitochondrial matrix is at the mM level; this means that the GTP produced in this process is around the mM level too.

The proton flux rate through the IV complex able to activate ATP synthase has been estimated as 3 × 10^21^ protons per second coupled with an ATP synthesis rate of 9 × 10^20^ molecules per seconds; to have an idea 65 kg ATP per day in resting conditions (ATP molecular mass is 507,18 g per mole), so the rate of GTP production could be at this extent and even more thinking about the metabolic engagement during sustained contraction (Zimmerman et al., 2011).

The cytosolic millimolar GTP concentration serves many physiological processes—to mention one of the most important, the G-protein coupled signal transduction and GTP-dependent enzymatic activity, but it may also serve as a sort of metabolic state. We have to consider that Guo is an excellent substrate for the human purine nucleoside phosphorylase that produces GMP, GDP, and GTP (Bzowska et al., 1990). However, the GTP mitochondrial metabolic production sustained by skeletal muscle contraction could determine a large amount of Guo saved in the cytosol and available for exosomal cargo.

### GTP and the metabolites BH4 and neopterin are important modulators of both skeletal muscle and the central nervous system signaling related to weakness and fatigability

Is it taken for granted that SM shows fatigue after prolonged contraction as during prolonged physical activity? Muscle weakness and fatigability could be caused by disorders of carbohydrate and lipid metabolism as for most metabolic myopathies (Gooch and DiMauro, 2003), phosphorylase deficiency, and ionic unbalance. However, in many subjects, symptoms of typical exercise intolerance, such as weakness and fatigue, seem nonspecific.

In this scenario, the role of GTP in the production of metabolites as tetrahydrobiopterin (BH4) and neopterin has been relatively neglected.

Among the functions already mentioned, iGTP is the enzymatic cofactor and substrate for guanosine triphosphate cyclohydrolase I (GTPCH1), the enzyme responsible for tetrahydrobiopterin (BH4) synthesis. BH4 is a cofactor of the phenylalanine hydroxylase that transforms the amino acid phenylalanine into tyrosine avoiding phenylalanine accumulation and the possible related metabolic disorder. In fact, BH4 deficiency is a rare and harmful metabolic disorder ranging from mild (low skeletal muscle tone) to severe symptoms (movement and cognitive disorders) that can affect people at different ages (Opladen et al., 2020). It is worth mentioning that BH4 also is involved in the production of monoamine neurotransmitters, the generation of nitric oxide, and pain (Werner et al., 2011; Latremoliere et al., 2015).

Physiological basal levels of BH4 are at tightly controlled concentrations, requiring a tuned regulation of BH4 synthesis. Considerably, different metabolic pathways (*de novo* synthesis, recycling, and salvage pathways) cooperate to maintain appropriate intracellular levels of BH4, but insufficient attention has been paid to the fundamental role of GTP and its effective concentration. In fact, the higher the GTP, the higher the BH4 production, in dependence of the GTPCH activity (for a review see Ghisoni et al., 2015b). As mentioned before, when mitochondrial metabolism, which in turn depends on skeletal muscle contraction engagement, does not sustain GTP production, probably BH4 level also could be impaired sustaining muscle fatigability. Moreover, GTPCH is an inducible enzyme, having its expression controlled by pro-inflammatory mediators, such as interferon-gamma (IFN-γ), tumor necrosis factor-alpha (TNF-α), interleukin-1beta (IL-1β), and lipopolysaccharides (LPS) (Werner-Felmayer et al., 1989), all mediators of skeletal muscle trophism.

Moreover, BH4 is crucial for the synthesis of the catecholaminergic neurotransmitters such as dopamine and serotonin. It is also mandatory for the cleavage of ether lipids as well as the biosynthesis of nitric oxide (ThöNy et al., 2000; Werner et al., 2011) to be useful for vasodilation (Ghisoni et al., 2015a; Ghisoni et al., 2015b; de Paula Martins et al., 2018; Latini et al., 2018). Altogether, these findings suggest muscle fatigability both at the peripheral and the central level.

## Discussion

The Guo and GTP molecules are present in the cytoplasm of resting cells at a concentration of hundred micromolars or millimolars, and the balance between them is tuned by purine nucleoside phosphorylase and nucleotidase activities.

At a glance, the skeletal muscle–central nervous system axis seems promising in terms of mutual influence and communication. In fact, thousands of studies claimed and demonstrated that physical exercise (physiologically sustained by mitochondrial-mediated metabolic activation of skeletal muscle contraction) positively influences mood and cognitive aspects. The main role seems to be tributed to myokines able to stimulate neurons (Severinsen and Pedersen, 2020). In this scenario, the Guo-based molecules can also act as regulators released and delivered through exosomes. Contracting skeletal fibers and their adult stem cells, satellite cells send Guo-based messages *via* exosomes, bringing positive regulator message to neurons and glia. This model is supported by the possibility of exosomes to cross the blood–brain barrier and subsequently affect brain processes (Saeedi et al., 2019).

The missing step in this speculative opinion remains in the investigation on Guo-exosome cargo and free Guo/GTP molecule uptake and release. Moreover, it remains unclear on how and how long the eGTP could be present in the extracellular space to initiate the signal transduction, or if endonucleotidases attack the eGTP to produce Guo in turn, which is the real responsible for the signal transduction.

It is worth mentioning that the Guo concentration into exosomes released by *in vitro* human satellite cells was about 5 mM (Pietrangelo et al., 2018). This sounds like a good cargo for shipment forwarded to the destination, and the central nervous system seems a suitable recipient for it. In fact, Guo-based purines have been recently proposed to be not only metabolic agents but also extracellular signaling molecules that regulate essential functions in the central nervous system, so it needs to be refurnished as much as possible, and skeletal muscle activity seems to be a very powerful source. In such a way, Guo-mediated neuroprotection, behavioral responses, and neuronal plasticity are based on skeletal muscle metabolism, specifically on mitochondrial-sustained contraction.

Guo-based exosomal cargo released by skeletal muscle could be considered God Hermes who brings its message: “movement is the essence of life and the essence of human health.” In this scenario, Guo-based molecules activate and tune the human physiology of excitable tissues.

## Key Concept

The key concept is that GTP mitochondrial production sustained by skeletal muscle contraction could determine tetrahydrobiopterin BH4 synthesis and a large amount of Guo saved in the cytosol and available for exosomal cargo. Guo-stuffed exosomes are delivered to neurons in the brain. Guo-based molecules are transformed into GTP and BH4, and in turn to several neurotransmitters. BH4 is involved in the production of catecholaminergic neurotransmitters such as dopamine and serotonin and the generation of nitric oxide. Briefly, Guo-based molecules activate and tune the human excitable tissues.
